# Morphologic characterization of residual DNA methylation in the gastric mucosa after *Helicobacter pylori* eradication

**DOI:** 10.1002/cam4.1082

**Published:** 2017-05-30

**Authors:** Sayumi Tahara, Tomomitsu Tahara, Tetsuya Tuskamoto, Noriyuki Horiguchi, Tomohiko Kawamura, Masaaki Okubo, Takamitsu Ishizuka, Mitsuo Nagasaka, Yoshihito Nakagawa, Tomoyuki Shibata, Makoto Kuroda, Naoki Ohmiya

**Affiliations:** ^1^ Department of Diagnostic Pathology I Fujita Health University School of Medicine Toyoake Japan; ^2^ Department of Gastroenterology Fujita Health University School of Medicine Toyoake Japan

**Keywords:** DNA methylation, gastric mucosa, *H. pylori* eradication, narrow‐band imaging endoscopy

## Abstract

Residual DNA methylation in the gastric mucosa after *Helicobacter pylori* (*H. pylori*) eradication may have a role in gastric carcinogenesis. We examined the association between morphologic features and promoter methylation status of non‐neoplastic gastric mucosa especially after *H. pylori* eradication. A total of 140 gastric specimens from 99 participants who had at least 6 months of post‐eradication period were examined. The magnifying narrow‐band imaging (NBI) endoscopic feature of gastric mucosa was divided into two types: restored‐small, round pits, accompanied with honeycomb‐like subepithelial capillary networks; atrophic‐well‐demarcated oval or tubulovillous pits with clearly visible coiled or wavy vessels. Methylation status of five candidate genes (*MYOD1*,* SLC16A12*,* IGF2*,* RORA*, and *PRDM5*) were examined by bisulfite pyrosequencing. The atrophic type, informative endoscopic features of intestinal metaplasia, demonstrated higher methylation levels in all five genes compared to the restored type (all *P *<* *0.0001). In the restored type, methylation levels were significantly lower among the samples with longer post‐eradication period (for all genes, *P *<* *0.0001), which was not observed in atrophic type (for all genes, *P *>* *0.1). Multivariate analysis demonstrated that atrophic type or presence of intestinal held an independent factor for hyper methylation (odds ratio: 24.69, 95% confidence interval: 6.95–87.76, *P *<* *0.0001). The atrophic type by the magnifying NBI and presence of intestinal metaplasia are the morphologic characteristics of residual DNA methylation of after *H. pylori* eradication, regardless of the post‐eradication period and it might be considered as the epigenetic irreversible point with *H. pylori* eradication.

## Introduction

Promoter CpG island methylation and subsequent transcriptional repression are important mechanisms in many types of cancers, while the aberrant methylation is also observed in non‐neoplastic tissues with aging and chronic inflammation 1, 2. *Helicobacter pylori* (*H. pylori*) infection plays an important role in gastric cancer development 3, 4, 5. *H. pylori*‐infected gastric mucosa is characterized as chronic inflammation and atrophy 5, leading to epigenetic changes characterized by the promoter methylation of multiple genes 6, 7. This phenomenon can be explained by the concept of an “epigenetic‐field‐defect” which is linked to gastric cancer predisposition.

Several studies have reported that *H. pylori* eradication prevents gastric cancer 8, 9, which might be attributed to the improvement of chronic inflammation or atrophy 10. The data also demonstrated that the therapy could reverse the hypermethylation in certain genes 11, 12. Even after successful *H. pylori* eradication, on the other hand, gastric cancers are sometimes identified 13, 14. The residual methylation in gastric mucosa after eradication might be relevant to this process 12, 15.

It is well known that the pathologic state of gastritis, such as severe gastric mucosal atrophy or intestinal metaplasia, is closely associated with the risk of gastric cancer among *H. pylori‐*infected patients 5. Recent studies have also suggested that the severe gastric atrophy after successful eradication is associated with increased risk of gastric cancer especially in gastric fundic area 16. We have reported that the morphologic appearances of gastric mucosa seen by the magnifying narrow‐band imaging (NBI) endoscopy are clearly distributed into atrophic or non‐atrophic mucosa, and correlating with gastric cancer occurrence after *H. pylori* eradication (Tahara et al., submitting). Various genotoxic and epigenetic changes in gastric mucosa which reflect past exposure of *H. pylori* infection might associate with the risk of gastric cancer after eradication. Morphologic identification of “field defect” has important implications for preventing gastric cancer. We examined the promoter methylation levels of candidate genes in non‐neoplastic gastric mucosa among patients with various post‐eradicated periods. We investigated the association among methylation status, magnifying NBI and histologic features.

## Materials and Methods

### Study population

Study participants were prospectively enrolled from patients attending the endoscopy Center of Fujita Health University from January 2013 to March 2016. A total of 99 participants were invited and all agreed to participate. These 99 participants had a history of successful *H. pylori* eradication therapy for the various reasons; gastric or duodenal ulcer, chronic gastritis or after endoscopic resection (ER) of early gastric cancer. For *H. pylori* eradication, triple therapy using one of following three regimens were used for all the participants. (1) 10 mg of rabeprazole sodium b.d., 200 mg of clarithromycin b.d., and 750 mg of amoxicillin b.d., (2) 30 mg of lansoprazole b.d., 200 mg of clarithromycin b.d., and 750 mg of amoxicillin b.d. or (3) 30 mg of lansoprazole b.d., 250 mg of metronidazole b.d., and 750 mg of amoxicillin b.d. For all the participants, success of *H. pylori* eradication was confirmed based on the C‐urea breath test at least 12 weeks after the triple therapy. Median period after eradication therapy was 41 months (ranging from 6 to 330 months). In all, 34 participants visited to our hospital for the treatment of early gastric cancer diagnosed after *H. pylori* eradication and seven of these cases were metachronous gastric cancers, diagnosed after ER and subsequent *H. pylori* eradication. A total of 23 healthy participants without history of *H. pylori* infection were also enrolled from patients who underwent upper gastroscopy for yearly checkup examination or for the complaints of abdominal discomfort (median age: 53 years, female/male, 11/12). Fujita Health University of Medicine approved the protocol, and written informed consent was obtained from all participants.

### Endoscopic procedure and sample collection

All participants underwent esophagogastroduodenoscopy (EGD) using a magnifying video endoscope (Olympus GIF‐H260Z and a CV260SL/CV290SL endoscopic system (Olympus Medical Systems). For the evaluation of endoscopic feature of gastric mucosa, the non‐pathologic mucosa of the gastric body was carefully evaluated with complete magnification coupled with a NBI light source.

**Figure 1:**
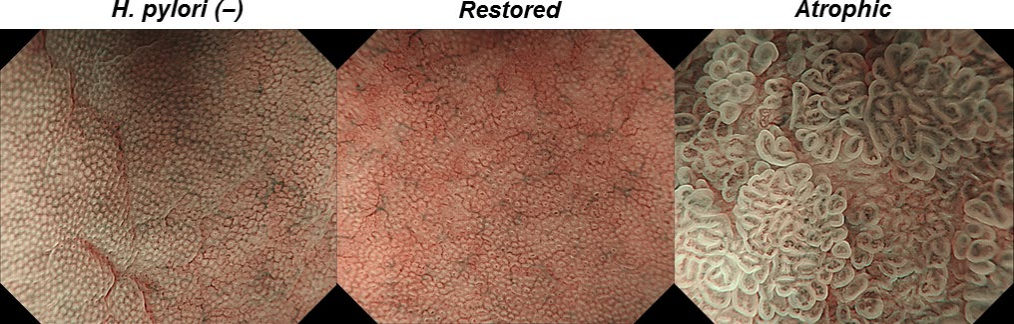
Magnifying NBI patterns of gastric body. Left, healthy gastric body without history of *Helicobacter pylori (H. pylori)* infection; Uniform small, round pits, accompanied with regular honeycomb‐like subepithelial capillary networks are seen. Center, restored type; small, round pits, accompanied with honeycomb‐like subepithelial capillary networks are also shown. Right, atrophic type; well‐demarcated oval or tubulovillous pits with clearly visible coiled or wavy vessels are seen.

The endoscopic feature of gastric mucosal morphology after *H. pylori* eradication has recently been reported (Tahara et al., submitting). The magnifying NBI patterns of gastric body can be divided into the following two types: restored and atrophic types (Fig. 1). The restored type is characterized as small, round pits, accompanied with honeycomb‐like subepithelial capillary networks, which resemble to healthy gastric body without history of *H. pylori* infection (Tahara et al., submitting). The atrophic type is characterized as well‐demarcated oval or tubulovillous pits with clearly visible coiled or wavy vessels, which is tightly linked to the presence of histologic intestinal metaplasia (Tahara et al., submitting). The classification of mucosal patterns among each case was based on the most predominant magnifying NBI pattern. The most predominant magnifying NBI pattern was taken as endoscopic pictures, and we obtained target biopsy specimens from that site. In case there were both restored and atrophic types in the same patient, endoscopic pictures and target biopsy specimens were obtained from both sites. At least, two biopsy specimens were obtained from targeted sites. We use one biopsy specimen to investigate the presence of intestinal metaplasia by the histologic analysis. The other biopsy was immediately frozen and stored at −80°C for the DNA methylation analysis. If there were obvious lesions, such as polyps, erosions, or cancers were seen, NBI scanning and target biopsy were performed getting enough distance from these pathologic lesions to avoid their influences. Endoscopic observations, classification of NBI patterns, and targeted biopsies were all performed by one expert endoscopist (T. T.). The interobserver concordance using representative endoscopic pictures from all participants demonstrated good *κ* coefficient values greater than 0.80 for both restored and atrophic patterns across an additional three expert endoscopists (M. O. N. H., and T. S., Tahara et al., submitting). Among 99 patients who have history of *H. pylori* eradication, one patient was taken five specimens, 30 patients were taken two specimens and 62 patients were taken one specimen for the DNA methylation analysis, respectively. One patient, who had taken annual follow‐up examination third times, was taken three specimens totally; two patients, who had taken annual follow‐up examination twice, was taken two specimens totally. One specimen was taken from each examination for the remaining patients. Thus, 140 biopsy specimens were obtained in total from 99 participants after *H. pylori* eradication. For the 23 healthy participants without history of *H. pylori* infection*,* one specimen was taken from each examination from greater curvature of uninvolved gastric corpus. Together, 163 biopsy specimens were stored for the molecular study.

### DNA methylation analysis by bisulfite pyrosequencing

DNA was extracted using the standard protein precipitation method. Bisulfite pyrosequencing was used to quantify the promoter methylation of five genes (*MYOD1*,* SLC16A12*,* IGF2*,* RORA*, and *PRDM5*). Their selection was based on the frequency of methylation in gastric cancer (*RORA* and *PRDM5*) 17 or *H. pylori*‐infected gastric mucosa (*MYOD1*,* SLC16A12*, and *IGF2*) 18. Methylation of all these genes were also well correlated with magnifying NBI features of *H. pylori*‐infected patients (Tahara et al., submitting).

Bisulfite‐treated genomic DNA was used to evaluate the methylation status by bisulfite pyrosequencing. Bisulfite treatment of DNA was carried out using an EZ DNA Methylation Kit (Zymo Research) according to the manufacturer's protocol. Pyrosequencing was carried out using a PSQ24 system with Pyro‐Gold reagent Kit (QIAGEN, Tokyo, Japan), and the results were analyzed using PyroMark Q24 software (QIAGEN). The primers used for pyrosequencing are listed in Table S1. To evaluate the quality of absolute methylation quantification of each assay, we used methylation positive and negative control DNAs. CpG Methylase (Ipswich, MA) treated genomic DNA was used as the methylation positive control and the genomic DNA amplified using GenomePlex^®^ Complete Whole Genome Amplification Kit (Tokyo, Japan) was used as the methylation negative control.

### Data analysis

Continuous variables between un‐matched two groups were assessed using the student's *t*‐test. Continuous variables between matched two groups were assessed using the Wilcoxon Signed Rank test. The correlation of continuous variables between two groups was assessed using the Spearman correlation analysis. An unsupervised hierarchical clustering analysis was used to identify distinct subgroups based on the methylation status of the selected five genes. Univariate and multivariate analyses were also performed to assess the factors related to DNA methylation. *P* value <0.05 was considered statistically significant.

## Results

### DNA methylation status of gastric mucosa, in relation to the magnifying NBI features

The clinic‐pathologic characteristics of 99 participants are shown in Table 1.

**Table 1:** Clinic‐pathologic characteristics of 99 participants

Variables	
Age: median (range)	68 (44–86)
Gender: male % (*n*)	68.7% (68)
Duration after *H. pylori* eradication: median months (range)	41 (6–360)
Reason for *H. pylori* eradication:	
Gastric or duodenal ulcer % (*n*)	23.2% (23)
After ER for early gastric cancer	27.3% (27)
Others	49.5% (49)
Gastric cancer occurrence	
Gastric cancer diagnosed before *H. pylori* eradication	27.3% (27)
Gastric cancer diagnosed after *H. pylori* eradication	34.3% (34)^a^
Cancer free	45.4% (45)

ER, endoscopic resection.

a For seven cases, metachronous gastric cancer was detected after subsequent *H. pylori* eradication after ER.

We analyzed the methylation levels of five genes (*MYOD1*,* SLC16A12*,* IGF2*,* RORA*, and *PRDM5*) in 140 gastric mucosa biopsies from 99 participants after *H. pylori* eradication. The five genes we analyzed are frequently methylated in gastric cancer (*RORA* and *PRDM5*) 10 or *H. pylori*‐infected gastric mucosa (*MYOD1*,* SLC16A12*, and *IGF2*) 11. Initially, we investigated the association between methylation status of those five genes and gastric mucosal morphologic features seen by the magnifying NBI endoscopy (Fig. 1).

**Figure 2:**
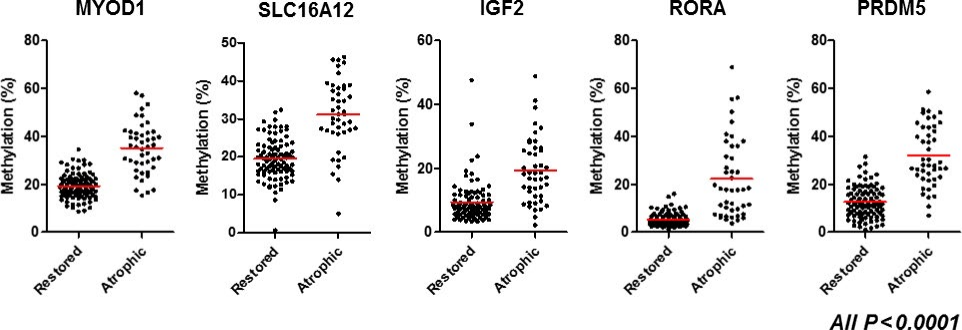
Methylation status of five gene promoters (*MYOD1*,* SLC16A12*,* IGF2*,* RORA*, and *PRDM5*) among restored and atrophic types. Horizontal bars represent mean methylation percentage. Statistical analysis was performed using Student's *t*‐test.

**Figure 3:**
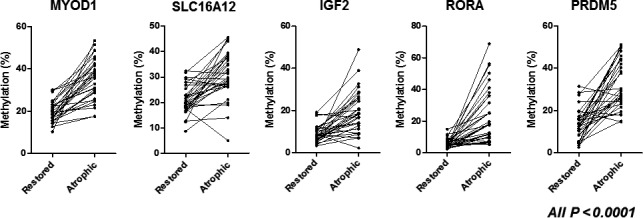
Methylation status of five gene promoters (*MYOD1*,* SLC16A12*,* IGF2*,* RORA*, and *PRDM5*) among restored and atrophic types among matched samples in patients who had both restored and atrophic types in the individual stomach. Statistical analysis was performed using Wilcoxon Signed Rank test.

We classified the 140 biopsy specimens into restored type (95 specimens) and atrophic type (45 specimens). We found that methylation levels of all five genes in the atrophic type were significantly higher than those of restored type (all *P *<* *0.0001: Fig. 2). We next compared the methylation levels of restored and atrophic types in 30 patients who had both restored and atrophic types in the individual stomach. Since one patient was taken five biopsies at endoscopic examination, 34 matched samples from these patients were included for this analysis. The significant higher methylation levels in the atrophic type were also confirmed in all five genes (all *P *<* *0.0001: Fig. 3).

### Association between DNA methylation and duration after *H. pylori* eradiation

**Figure 4:**
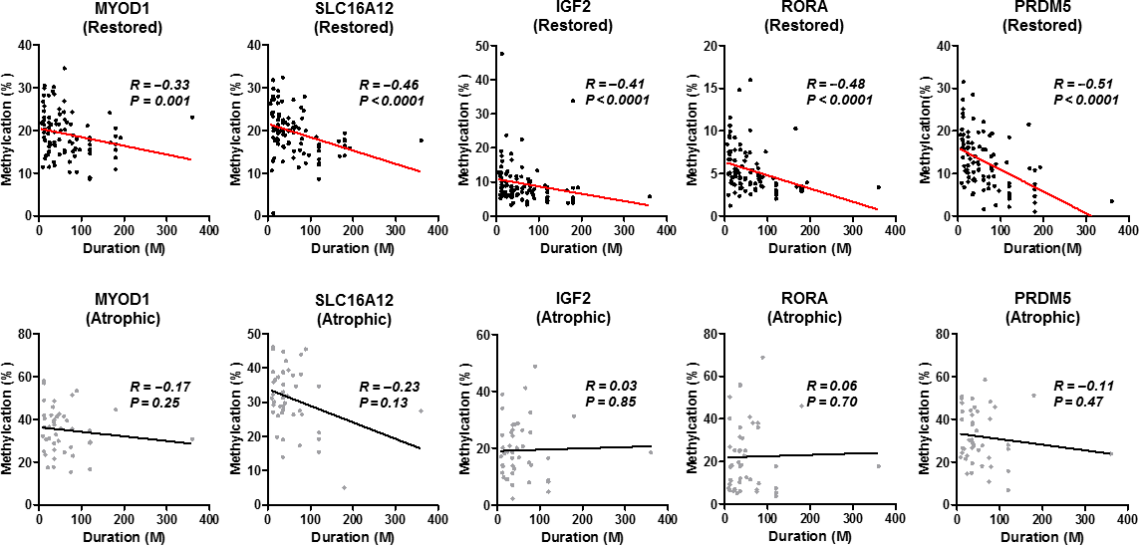
Association between methylation status and duration after *H. pylori* eradication among five gene promoters (*MYOD1*,* SLC16A12*,* IGF2*,* RORA*, and *PRDM5*). Upper, restored type; Lower, atrophic type; Statistical analysis was performed using Spearman correlation analysis.

DNA methylation levels in the gastric mucosa improved after *H. pylori* eradiation by the time‐dependent manner 12. We next investigated the association between methylation levels and post‐eradication period. In the restored type, methylation levels were significantly decreased in patients with longer post‐eradication period (all *P *<* *0.0001, Fig. 4). We also compared the methylation status of five genes in the restored type and the 23 healthy gastric mucosae without history of *H. pylori* infection. For three genes (*IGF2*,* RORA*, and *PRDM5*), DNA methylation levels of restored type after 10 years of post‐eradication period were similar to those of the healthy gastric mucosa without *H. pylori* infection (all *P *>* *0.1), while it remained higher for two genes (*MYOD1*,* SLC16A12*, both *P *<* *0.0001, Fig. S1). Concerning the atrophic type, we did not observe any correlation between methylation status of all five genes and duration after *H. pylori* eradiation. Moreover, the correlation analysis of methylation status between restored and atrophic types among patients who had both restored and atrophic types in the individual stomach only showed significant correlation only in one gene (*MYOD1*,* P *=* *0.03, Fig. S2). It is suggested that gastric mucosa has renewal capacity regarding the methylation levels in the restored type, but it would be lost when it turned into the atrophic type.

### Evaluation of clinic‐pathologic factors in relation to the methylation status in the gastric mucosa after *H. pylori* eradication

**Figure 5:**
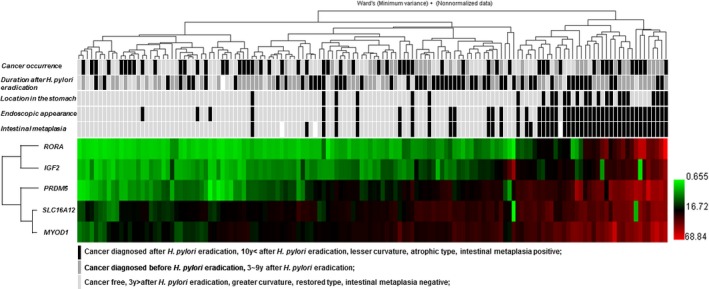
Unsupervised hierarchical clustering analysis of five gene promoters (*MYOD1*,* SLC16A12*,* IGF2*,* RORA*, and *PRDM5*) among 140 samples. Black, cancer diagnosed after *H. pylori* eradication, 10 years < after *H. pylori* eradication, lesser curvature, atrophic type, intestinal metaplasia positive; gray, cancer diagnosed before *H. pylori* eradication, 3–9 years after *H. pylori* eradication; light gray, cancer free, 3 years > after *H. pylori* eradication, restored type, intestinal metaplasia negative; white, not determined; Note that seven metachronous gastric cancer cases diagnosed after endoscopic resection and subsequent *H. pylori* eradication were treated as cancer diagnosed after *H. pylori* eradication.

We investigated association between the clinic‐pathologic factors and the methylation status in the gastric mucosa after *H. pylori* eradication. We performed unsupervised hierarchical clustering analysis based on the methylation status of the selected five genes (Fig. 5). This analysis demonstrated that the atrophic types were mainly clustered as hypermethylated samples. The samples with intestinal metaplasia were also clustered as hypermethylated samples and tightly linked to the atrophic type, which was in line with recent our study (Tahara et al., submitting). We also found that the samples obtained from lesser curvature were clustered as hypermethylated samples. On the other hand, there was no clear association between cancer occurrence, duration after *H. pylori* eradication and the methylation status. We performed univariate and multivariate analyses to assess the factors related to DNA methylation statistically. We calculated mean *Z*‐score methylation of five genes to define hypermethylated samples. The mean *Z*‐score methylation of all five PCGIs in the gastric mucosa presented an approximately Gaussian distribution, with over represented of methylation‐high cases, we set cutoff value of 0.006 (mean *Z*‐score methylation) for the definition of methylation‐high cases. Univariate analysis revealed that location (lesser curvature, odds ratio: 13.05, 95% confidence interval: 4.16–40.92, *P *<* *0.0001), longer duration after *H. pylori* eradication (odds ratio: 0.99, 95% confidence interval: 0.98–1.00, *P *=* *0.016), and presence of atrophic type or intestinal metaplasia (odds ratio: 64.92, 95% confidence interval: 10.00–26.36, *P *<* *0.0001) were significantly associated with methylation‐high (Table 2). Multivariate analysis of these factors revealed that longer duration after *H. pylori* eradication significantly reduced the risk of DNA methylation (odds ratio: 0.99, 95% confidence interval: 0.98–1.00, *P *=* *0.019), and the presence of atrophic type or intestinal metaplasia held a strong risk for DNA methylation (odds ratio: 24.69, 95% confidence interval: 6.95–87.76, *P *<* *0.0001) as an independent factor.

**Table 2:** Univariate analysis assessing the factors related to methylation‐high

Variables	Odds ratio (95% confidence interval)	*P* value
Age	1.04 (0.99–1.08)	0.12
Gender (male)	1.69 (0.76–3.80)	0.2
Location (lesser curvature)	13.05 (4.16–40.92)	<0.0001
Duration	0.99 (0.98–1.00)	0.016
Cancer occurrence	1.97 (0.96–4.06)	0.066
Atrophic type or IM positive	64.92 (10.00–25.35)	<0.0001

IM, intestinal metaplasia.

Methylation‐high, mean *Z*‐score methylation > −0.006.

**Table 3:** Multivariate analysis assessing the factors related to methylation‐high

Variables	Odds ratio (95% confidence interval)	*P* value
Location	1.30 (0.26–6.52)	0.75
Duration	0.99 (0.98–1.00)	0.019
Atrophic type or IM positive	24.69 (6.95–87.76)	<0.0001

IM, intestinal metaplasia.

Methylation‐high, mean *Z*‐score methylation > −0.006.

## Discussion

We showed that the methylation status of gastric mucosa after *H. pylori* eradication is closely associated with its endoscopic and histologic features. The atrophic type, which is informative magnifying NBI appearance of intestinal metaplasia, demonstrated significantly higher methylation levels in all five genes, compared to the restored type.

The five genes analyzed in this study are frequently methylated in gastric cancer (*RORA* and *PRDM5*) 17 or *H. pylori*‐infected gastric mucosa (*MYOD1*,* SLC16A12*, and *IGF2)* 18. Methylation of all these genes are also well correlated with magnifying NBI and histologic features of *H. pylori*‐infected gastric mucosa (Tahara et al., submitting). DNA methylation after *H. pylori* eradication is thought to be associated with gastric cancer predisposition 15, but the morphologic features of this residual methylation have not been clearly described. Our current result provided the evidence that morphologic features related to the intestinal metaplasia are associated with residual methylation in gastric mucosa after *H. pylori* eradication. We classified the morphologic features of gastric body (fundic area) using magnifying NBI endoscopy, assuming that the inter‐individual difference in atrophic status after *H. pylori* eradication would be more enhanced in the gastric body rather than in the pyloric region (antrum). We have shown that the incidence as well as the portion of atrophic area were not associated with post‐eradication period, while the spread of atrophic area was an indicator for gastric cancer occurrence (Tahara et al., submitting). Other studies have also suggested that the severe gastric atrophy especially in the fundic area might be a risk of gastric cancer 16. The atrophic type with hyper‐DNA methylation in the gastric fundic area might reflect the past exposure of *H. pylori* infection. Several studies showed that *H. pylori* eradication could reverse DNA methylation by time‐dependent manner 12, 15. In fact, among restored type, methylation levels were significantly lower in patients with longer post‐eradication period in this study. The methylation levels of *IGF2*,* RORA*, and *PRDM5* genes among restored type with more than 10 years of post‐eradication period demonstrated almost same as healthy gastric mucosa without history of *H. pylori* infection. On the other hand, this issue was not applied to the atrophic type; there was no association between the duration and methylation levels. The atrophic type also presented significantly higher methylation levels comparing to the restored type in all genes among matched samples from patients who had both restored and atrophic types in the individual stomach, but they had no correlation with each other except *MYOD1* gene. This suggests that the atrophic type represents the epigenetic irreversible point with *H. pylori* eradication.

To evaluate the association between DNA methylation status and several clinic‐pathologic factors, we performed unsupervised hierarchical clustering analysis and multivariate analysis. Atrophic type was very tightly linked with intestinal metaplasia and clustered as hypermethylated samples. The multivariate analysis showed that atrophic type or intestinal metaplasia was the strong independent risk for DNA methylation after *H. pylori* eradication. Although DNA hypermethylation might be a risk of gastric cancer regardless of *H. pylori* infection status 6, 19, 20, 21, in our study, the most important factor of DNA hypermethylation was magnifying NBI and histologic features. This indicates that the methylation change is closely linked to the magnifying NBI and histologic features within the focal points. It might also be informative for estimating risk of gastric cancer to assess the spread of NBI patterns in the stomach (Tahara et al., submitting). Morphologic features of aberrant methylation have described as reflecting its distinct clinic‐pathologic features in colorectal cancer [22]. Our current result also demonstrated the reliability of the magnifying NBI features of gastric mucosa after *H. pylori* eradication to estimate the “field defect” from a molecular view point. Gastric cancer incidence after *H. pylori* eradication is increasing recently, but it is difficult to find it because of its ambiguity. 14. Our result might provide more appropriate clinical implementation for gastric cancer surveillance after eradication, reflecting individual cancer risk. We believe that our findings provide salient information for many endoscopists to further improve gastric cancer risk stratification among patients after *H. pylori* eradication.

## Conflict of Interest

The authors declare that they have no conflicts of interest.

## Supporting information


**Table S1.** Primer sequences used for pyrosequencing.
**Figure S1.** Methylation status of five gene promoters (*MYOD1*,* SLC16A12*,* IGF2*,* RORA* and *PRDM5*) among restored type and healthy gastric mucosa without history of *H. pylori* infection. Statistical analysis was performed using Student's *t*‐test.
**Figure S2.** Correlation of methylation status of five gene promoters (*MYOD1*,* SLC16A12*,* IGF2*,* RORA* and *PRDM5*) among restored and atrophic types in matched samples in patients who had both restored and atrophic types in the individual stomach. Statistical analysis was performed using Spearman correlation analysis.
