# Supplementary material for: Morphologic characterization of residual DNA methylation in the gastric mucosa after Helicobacter pylori eradication
Source: Cancer Med. 2017 May 30;6(7):1730–7. doi: 10.1002/cam4.1082 (PMC5504328; doi:10.1002/cam4.1082)
Supplement: Supplementary file 1 — Table S1. Primer sequences used for pyrosequencing. Figure S1. Methylation status of five gene promoters (MYOD1, SLC16A12, IGF2, RORA and PRDM5) among restored type and healthy gastric mucosa without history of H. pylori infection. Statistical analysis was performed using Student's t‐test. Figure S2. Correlation of methylation status of five gene promoters (MYOD1, SLC16A12, IGF2, RORA and PRDM5) among restored and atrophic types in matched samples in patients who had both restored and atrophic types in the individual stomach. Statistical analysis was performed using Spearman correlation analysis. [file CAM4-6-1730-s001.docx]

**Supplementary Tables and Figures**

**Supplementary Table 1.** Primer sequences used for pyrosequencing

| Assay name | Forward primer | Reverse primer (1st step PCR) sequence | Forward primer | Reverse primer (2nd step PCR) sequence | Sequencing primer sequence |
| --- | --- | --- | --- | --- | --- |
|  | (1st step PCR) sequence |  | (2nd step PCR) sequence |  |  |
| *MYOD1* | AATTAGGGGATAGAGGAGTATTGAAAG | ACAACCCTAAACRACTACACTTAACTC | GAAAGTTAGTTTAGAGGTGA | U-ACAACCCTAAACRACTACACTTAACTC | GAGGTTTGGAAAGGG |
| *SLC16A12* | TAGAGGGAGAGGTGGTTTAGGTGAT | CACCCAAATTAAAATCCCAAACTC | TAGAGGGAGAGGTGGTTTAGGTGAT | U-CACCCAAATTAAAATCCCAAACTC | AAGGGTATTTTTTAAGGAAG |
| *IGF2* | GAGGATTAGGGAGGGAAATATAGT | CCCAAACCCCCAAATTATC | GAGGATTAGGGAGGGAAATATAGT | U-CCCAAACCCCCAAATTATC | AATGGTTATTTAGTTTTTAG |
| *RORA* | TTTGGTATTATAGAGTTGTTTTGAAAATAGAA | ACCCAAACTAACTCCATATTTTTTCC | TTTGGTATTATAGAGTTGTTTTGAAAATAGAA | U-ACCCAAACTAACTCCATATTTTTTCC | TGAAAATAGAAGATAGAGGGA |
| *PRDM5* | TGAGGTTTTGGGGTTAGTTT | CRAATCCRTTCCTACCATTC | TGAGGTTTTGGGGTTAGTTT | U-CRAATCCRTTCCTACCATTC | GTTAATTTYGGGTTAATTAG |

U =biotin labeled universal primer tag: 5'-biotin-GGGACACCGCTGATCGTTTA


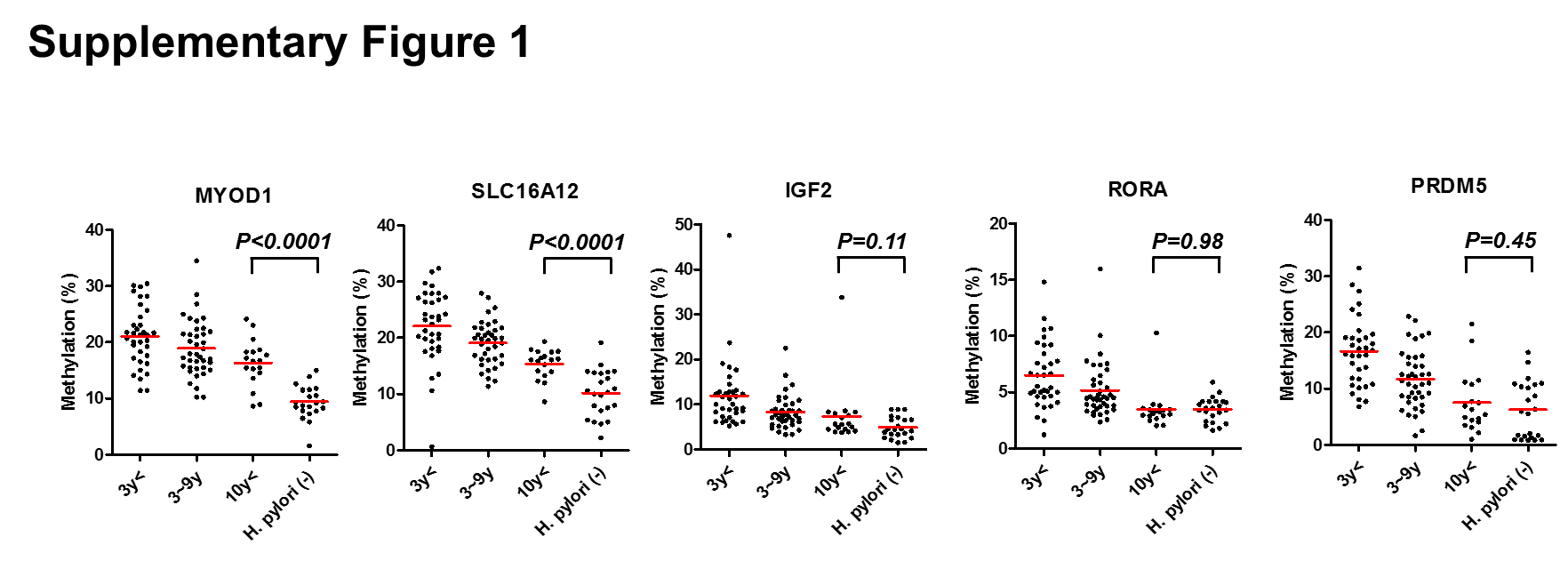


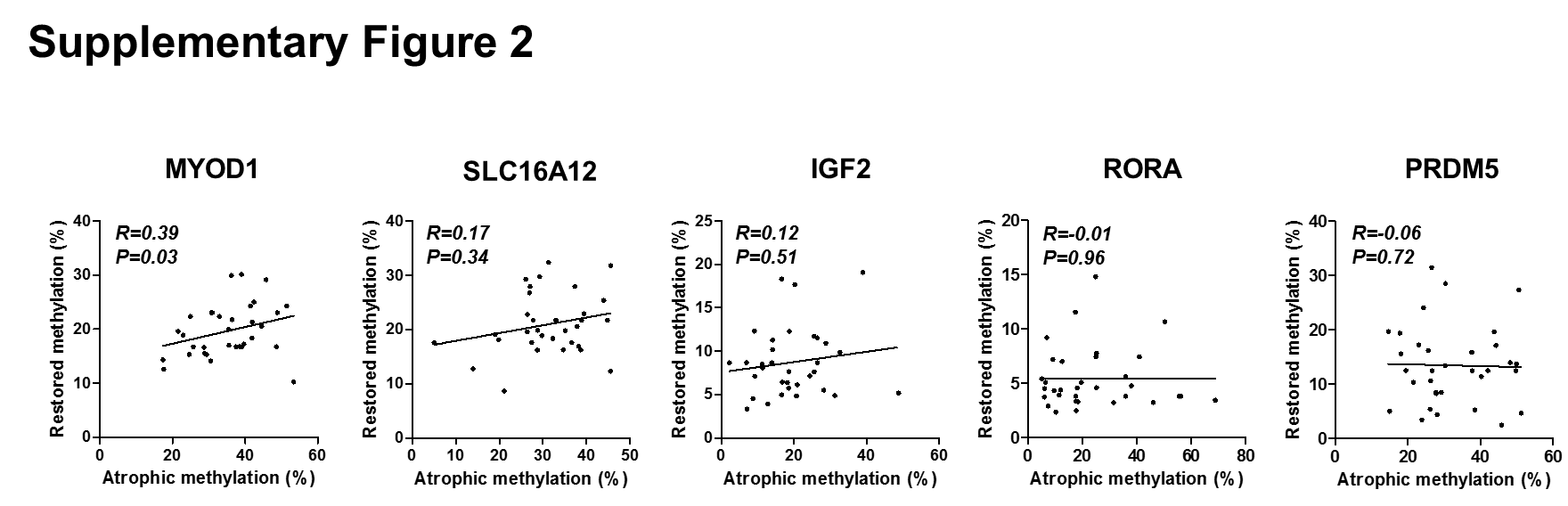


Supplementary figure 1

Methylation status of 5 gene promoters (*MYOD1*, *SLC16A12*, *IGF2*, *RORA* and *PRDM5*) among restored type and healthy gastric mucosa without history of *H. pylori* infection. Statistical analysis was performed using Student’s t-Test.

Supplementary figure 2

Correlation of methylation status of 5 gene promoters (*MYOD1*, *SLC16A12*, *IGF2*, *RORA* and *PRDM5*) among restored and atrophic types in matched samples in patients who had both restored and atrophic types in the individual stomach. Statistical analysis was performed using Spearman correlation analysis.
